# Evaluation of estimated daily intake (EDI) of cadmium and lead for rice (*Oryza sativa L.*) in calcareous soils

**DOI:** 10.1186/1735-2746-10-28

**Published:** 2013-04-08

**Authors:** Ali Chamannejadian, Gholamabbas Sayyad, Abdolamir Moezzi, Alireza Jahangiri

**Affiliations:** 1 Department of Soil Science, College of Agriculture, Shahid Chamran University of Ahvaz, Ahvaz, Khuzestan province, Iran; 2 Nanotechnology Research Center and Department of Medicinal Chemistry, School of Pharmacy, Ahvaz Jundishapur University of Medical Sciences, Ahvaz, Iran

**Keywords:** Cadmium, Lead, EDI, Rice, Calcareous soils

## Abstract

The excessive amounts of cadmium and lead in food chain can cause health problems for humans and ecosystem. Rice is an important food in human diet. Therefore this study was conducted in order to investigate cadmium and Lead concentrations in seed rice (Oryza saliva) of paddy fields in southwest of Iran. A total of 70 rice seed samples were collected from paddy fields in five regions of Khuzestan province, Southwest Iran, during harvesting time. In the samples cadmium and Lead concentrations were measured. To assess the daily intake of Cadmium and Lead by rice, daily consumption of rice was calculated. The results showed that average concentrations of Cadmium and Lead in rice seeds were 273.6 and 121.8 μg/kg, respectively. Less than 72% of rice seed samples had Cadmium concentrations above 200 μg/kg (i.e. Guide value for cadmium); and less than 3% had Lead concentrations above 150 μg/kg (i.e. Guide value for Lead). The estimated daily intakes of cadmium by the local population was calculated to 0.59 μg/day kg bw, which corresponds to 59% of the tolerable daily intakes (i.e. 1 μg/day kg bw). Eleven out of 70 samples (15.71%) exceed the tolerable daily intakes. The dietary intakes for Lead in the local population ranged from 0.22 to 0.47 μg/day kg bw. Tolerable daily intakes for Lead is 3.6 μg/day kg bw. As a whole, long term consumption of the local rice may bear high risk of heavy metal exposure to the consumer in the study region.

## Introduction

Heavy metals due to poisonous, accumulating traits and long longevity in organism’s body are considerably important. Many countries were infected to water and soil pollution crisis to heavy metals [1]. Human beings cause this contamination type through different ways. For example, applications of metal-contaminated fertilizers, animal manures, and sewage sludge can result in high concentrations of heavy metals in agricultural soils [2].

Plants are the main way of cadmium (Cd) and lead (Pb) transfer from contaminated soils to humans. In a Cd and Pb contaminated soil, plants can uptake more heavy metal and accumulate it in different organs especially edible parts [3]. This phenomenon is especially important for high consumption crops like rice. Rice is the dominant staple food crop in developing countries (including Iran) so that 96% of the world’s rice is produced and consumed in developing countries [4], making up over 70% of the daily energy intake [5]. The protein component in rice (7–9% by weight) is relatively low [6], but it forms a major source of protein (50%) in these countries [5]. Also rice is the second high consumption food among Iranian people. It is the most common crop grown in agricultural lands in the north of Iran [7].

In total, averages normal value of Cd and Pb concentrations in rice grains are 60 and 440 μg/kg, respectively [8-11]. Al-Saleh and Shinwari (2001), noted that the average levels of Cd and Pb in rice grains were 20 and 135 μg/kg, respectively [12,13]. Shimbo et al. (2007) also reported that the geometric mean contents of rice produced in Japan in 1998–2000 were 50 and 2 μg/kg based on fresh weight for Cd and Pb, respectively [1,5,6,9,10,14-16]. According to Bennett et al. (2000), the median values in wild rice seed from northern Wisconsin, USA, were 16 and 250 μg/kg for Cd and Pb, respectively [17-19]. Jung (1995) also reported that Cd and Pb concentrations in rice grown in various countries were in the range of 10–50 and 1–500 μg/kg, respectively [20].

Estimated daily intake (EDI) as a common index for metal transfer from plant to humans were calculated and used for rice in some studies [3,6,8,19,21,22].

Although several studies were conducted about Cd and Pb concentrations in rice grains, however to our knowledge, up to now few studies have investigated Cd and Pb concentrations in rice grain in calcareous soils where metals were supposed to be less available for plants than other soils. Previous researches showed that in calcareous soils, Cd and Pb solubility and also plant availability decreased due to metal-carbonate precipitation in higher pH, and calcium competition with metal cations for plant uptake [1,3-11,13-19,21-32]. Another problem in previous studies is the scale of the studies. Most previous studies were conducted in pot or small scale areas like experimental plots or small regions. The results of pot and hydroponic experiments may not predict the uptake of heavy metal by crops in field conditions. In addition, small scale studies could not into account spatial variability of soil properties and metal concentrations in soil and plants, which is very important in real field condition. Therefore this study was conducted in large scales areas where the mentioned issues were considered.

Cadmium and Pb were chosen for risk assessment because of their high toxicities or comparatively high levels in all of the collected rice samples in previous studies. Cadmium is toxic to the kidney and has a long biological half-life in human. Lead has shown to be associated with damnification of central nervous system [18]. It is therefore necessary to determine the dose level for human, which is considered to be taken daily over a lifetime without adverse effect.

The objectives of this large scale study were to 1) determine Cd and Pb concentrations of rice grain of paddy fields with calcareous soils; and 2) assess Cd and Pb intake from rice based on daily intake.

## Materials and methods

### Study area

**Figure 1:**
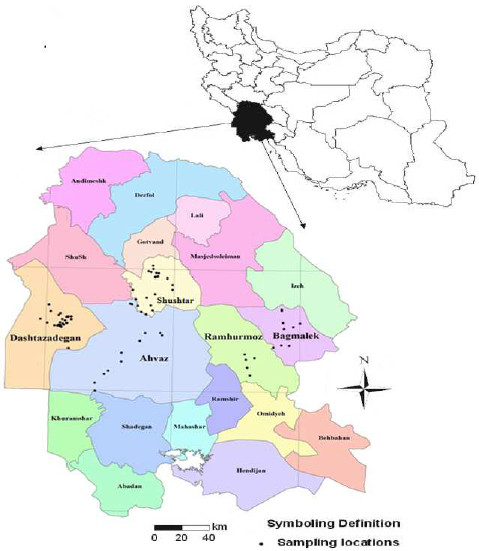
Distribution of sampling locations.

The study area was about 300 km^2^ based on distribution of rice fields in Khuzestan province, Southwest Iran, as shown in Figure 1. The study region consists of five sub regions including Dashtazadegan, Ahvaz, Shushtar, Ramhurmoz and Baghmalek (Figure 1). In total 70 paddy fields were randomly selected in study region and rice seed samples were taken. Statistically at least 30 points are suitable for tracing of a trend therefore 70 points are suitable for this large scale study for evaluating metal concentration in rice seeds. Similar researchers such as Zazuli et al. (2008), and Fuj et al. (2008) [1,3-10,14,15,19,22,24-28], also used the same number of sampling points for their large scale studies.

### Seed sampling and analysis

A total of 70 rice seed samples were collected from paddy fields in summery 2010. All sample sites were recorded using a hand-held Global Position System (GPS). The samples were obtained using a plastic spade to avoid any heavy metal contamination. The samples were oven-dried at 70°C to constant weight, followed by acid-digestion according to the AOAC method using HNO and HClO (25:10 ml) acids [23]. The clear digested liquid was filtered through a 0.45 μm acid-resistant filter paper. In filtered solution Pb and Cd concentrations were measured by an inductively coupled plasma mass spectrometer (ICP-MS Model HP4500).

### Tolerable daily intake (TDI) of Cd and Pb

The provisional tolerable weekly intake (PTWI) [1,3-11,14-19,22-34], recommended by the Joint FAO/WHO Expert Committee on Food Additives (JECFA), show appropriate safe exposure levels and is used to estimate the amount of contaminants, ingested over a lifetime without appreciable risk. The TDI of toxic metals such as Cd and Pb in this study was calculated according to the guide values suggested by Institute of Standard and Industrial Research of Iran (ISIRI). Based on ISIRI, the tolerable daily intakes of Pb and Cd through rice consumption was estimated at 1.0 and 3.6 μg/day kg bw, respectively.

### Estimated daily intake (EDI) of Cd and Pb through rice consumption

The daily intake of metals depends on both the metal concentration in food and the daily food consumption. In addition, the body weight of the human can influence the tolerance of contaminants. The EDI are a concept introduced to take into account these factors. The EDI is calculated as follows:

(1) $$\mathrm{EDI}=\frac{C\times \mathrm{Cons}}{\mathrm{Bw}}$$

Where C is the concentration of the heavy metals in contaminated rice; Cons stands for the daily average consumption of rice in the study region; and Bw represents the body weight. Based on the dietary nutrition intake level survey by Zhong et al., and Yu-sheng et al. [11,16,29-34], rice was the staple food for daily consumption, and the adult residents in the region had an average daily intake of 130 g rice per day. Their body weight was set to 60 kg in this study [6].

### Statistical analysis

All the results were expressed on a dry weight basis. All statistical analyses were performed with SPSS 16.0 for Windows.

## Results and discussion

The results of cadmium and lead contents in 70 samples of rice seed from five subregions are shown in Table 1. The concentrations of Cd and Pb in rice seed samples were in the range of 63.3–521 and 100–218.8 μg/kg, respectively. The maximum concentration of Cd was 521 μg/kg in Ahvaz sub region. The mean concentration of Cd and Pb were 273.6 and 121.8 μg/kg, respectively, which were greater than (for Cd) or near to (for Pb) the ISIRI standards for Cd (i.e. 0.2 mg/kg) and Pb (i.e. 0.15 mg/kg) in rice seed. More than 71 percentages of rice seed samples had Cd content greater than ISIRI standard. While 3 percentages of samples had Pb contents greater than ISIRI standard. Therefore inhabitants in the study region who consume rice are more expose to Cd contamination risk than Pb.

**Table 1:** The mean and range concentrations (μg/kg) of the studied rice sample

**Element**	**Area**	**N**	**Mean ± SD**	**Min**	**Max**
**Cd**	**Ahvaz**	12	270.9 ± 128	120	521
	**Baghmalek**	9	296.7 ± 135	127	465
	**Dashtazadegan**	24	275.5 ± 118	63.3	493
	**Ramhurmoz**	5	249.6 ± 24.0	219	283
	**Shushtar**	20	269.6 ± 123	97	515
	**Total**	70	273.6 ± 117	63	521
**Pb**	**Ahvaz**	12	115.9 ± 6.0	105	128
	**Baghmalek**	9	131.8 ± 34	109	219
	**Dashtazadegan**	24	120.6 ± 11	105	150
	**Ramhurmoz**	5	123.2 ± 1.0	121	124
	**Shushtar**	20	122 ± 10.0	100	145
	**Total**	70	121.8 ± 15.0	100	219

The comparison of EDI with the respective TDI for Cd and Pb are shown in Table 2. It was assumed that the local population consumes the local rice, and the EDI that was calculated from Eq. (1) is based on heavy metal levels from the rice samples. The EDI of Cd by the local population was calculated to 0.59 μg/day kg bw, which corresponds to 59 percentage of the TDI (1 μg/day kg bw). The maximum daily intake of Cd from rice was 1.13 μg/day kg bw, which calculated from the maximum concentrations of Cd in rice in Ahvaz, and was 0.13-fold greater than TDI. Eleven out of 70 samples (15.47%) exceed the TDI. On the other hand, some individuals in this area may consume more than twice of the average amount of rice and their daily dietary intakes of Cd would further exceed the TDI. The TDI for Pb was set by the National Nutrition and Food Research Institute of Iran at 3.6 μg/day kg bw. The dietary intakes for Pb in the local population ranged from 0.22 to 0.47 μg/day kg bw with a mean value of 0.26 ± 0.03 μg/day kg bw. The maximum daily intake of Pb from rice was 0.47 μg/day kg bw which calculated from the maximum concentrations of Pb in rice in Baghmalek, which was less than TDI. It is important to note that the calculated EDI in this study for Pb (which is less than TDI) was only obtained through rice consumption, and including Pb intake through dietary would probably increase the EDI values. Some researchers also reported similar amounts of EDI of Cd and Pb through rice consumption [1]. Investigation of Cd content of rice from different countries revealed a range of 0.0008 to 0.13 mg/kg with the average of 0.03 mg/kg. The mean Cd content values in rice seeds reported for Japan were 50 ng/kg dry wt in 1998–2000 and 0.01 mg/kg dry wt for Taiwan in 2004 [1,5,6,9-11,15,16,29-32]. The studies in other parts of Iran showed the different ranges of Cd in rice seeds. Afshar et. al. (2000) measured mean Cd concentration in Amol rice (an Iranian rice variety) 0.09 mg/kg [29].

**Table 2:** Mean EDI by a 60 kg body weight person and the range in Khuzestan

**Element**	**Area**	**N**	**Mean ± SD**	**Min**	**Max**
**Cd**	**Ahvaz**	12	0.59 ± 0.28	0.26	1.13
	**Baghmalek**	9	0.64 ± 0.29	0.28	1.01
	**Dashtazadegan**	24	0.59 ± 0.30	0.14	1.07
	**Ramhurmoz**	5	0.54 ± 0.05	0.47	0.61
	**Shushtar**	20	0.58 ± 0.27	0.21	1.11
	**Total**	70	0.59 ± 0.25	0.14	1.13
**Pb**	**Ahvaz**	12	0.25 ± 0.01	0.23	0.28
	**Baghmalek**	9	0.29 ± 0.07	0.24	0.47
	**Dashtazadegan**	24	0.26 ± 0.02	0.23	0.33
	**Ramhurmoz**	5	0.27 ± 0.00	0.26	0.27
	**Shushtar**	20	0.26 ± 0.02	0.22	0.32
	**Total**	70	0.26 ± 0.03	0.22	0.47

The studies of Khani and Malekoti (2000 a, b) showed that averaged Cd content in rice produced in north of Iran was 0.34 mg/kg with a range of 0.25-0.45 mg/kg [26,27]. The authors also showed that Cd content of soil increased gradually from 33 mg/kg in 1998 to 34 mg/kg in 1999. They reported that with increasing rice consumption, this level of Cd content of rice seeds can easily pose a great threat for human health.

The reasons for higher Pb and Cd contents in rice are complex. Greater Pb and Cd in rice seeds are mostly due to high metal concentrations in contaminated soil. Chamannejadian et al. reported high Cd concentration in the rice and soils of Khuzestan province [18]. In a contaminated soil, the availability of metal for plant uptake increased. In addition, Cd and Pb uptake by rice is a function of different soil and plant characteristics like soil pH, soil organic matter, clay percentage, soil salinity, chloride concentration, carbonate, lime; and rice species [8,15,33]. Metal solubility is known to increase with a decrease in soil pH and hence plant metal uptake is higher in acidic soils. Therefore, any reduction in soil pH in these farms could raise metal availability and metal uptake by plants, which ultimately could increase health risk. It is also known that there is a linear relationship between metal availability and organic matter content. In addition absorption of heavy metal from soil to the rice seed can extensively be modified by the redox potential of the soil, which is affected by the degree of water which covers the paddy fields during cropping season [2,20,33,34]. The soil and plant characteristics are different in the various subregions so the metal concentrations in rice seeds were different [17,25,28,29].

## Conclusion

The results of this study showed the roll of rice seeds on transferring Cd and Pb from soil to humans. In more than 70% of rice seed samples, Cd concentration was more than guide value of National Nutrition and Food Research Institute of Iran. However there is no serious rice contamination to Pb in the study area. In regards to the national food safety criteria, Pb content in few rice samples exceeded the National Nutrition and Food Research Institute of Iran. By estimating the daily intake of Pb and Cd by the local inhabitants, we concluded that the Cd daily intake in this area might exceed the TDI recommended by National Nutrition and Food Research Institute of Iran. Although the mean estimated daily intake of Cd from rice is 59% of the TDI, it still holds a high proportion of TDI, suggesting local rice consumption may induce excessive Cd intake as well. Also, Pb contamination through rice should not be neglected, though its mean EDI were lower than TDI. As a whole, long term consumption of the local rice may bear high risk of heavy metal exposure to the consumer. Moreover, a great deal of attention should also be paid regarding the contamination of biota through the food Khuzestan. Relevant data are still limited and further studies need to be conducted.

## Competing interests

The authors declare that they have no competing interests.

## Authors’ contributions

AC: conception and design, generation of data, collection of data, assembly of data, analysis of data, interpretation of data, drafting of the manuscript. GS: conception and design, interpretation of data, revision of the manuscript, approval of the manuscript. AM: interpretation of data, revision of the manuscript, approval of the manuscript. AJ: generation of data, revision of the manuscript, approval of the manuscript. All authors read and approved the final manuscript.
